# Modified combination of anti-thymocyte globulin (ATG) and post-transplant cyclophosphamide (PTCy) as compared with standard ATG protocol in haploidentical peripheral blood stem cell transplantation for acute leukemia

**DOI:** 10.3389/fimmu.2022.921293

**Published:** 2022-08-05

**Authors:** Maryam Barkhordar, Amir Kasaeian, Ghasem Janbabai, Hossein Kamranzadeh Fumani, Sahar Tavakoli, Amir Abbas Rashidi, Seied Asadollah Mousavi, Ardeshir Ghavamzadeh, Mohammad Vaezi

**Affiliations:** ^1^ Hematology, Oncology and Stem Cell Transplantation Research Center, Research Institute for Oncology, Hematology and Cell Therapy, Tehran University of Medical Sciences, Tehran, Iran; ^2^ Digestive Diseases Research Center, Digestive Diseases Research Institute, Tehran University of Medical Sciences, Tehran, Iran; ^3^ Inflammation Research Center, Tehran University of Medical Sciences, Tehran, Iran; ^4^ Cancer & Cell Therapy Research Center, Tehran University of Medical Sciences, Tehran, Iran

**Keywords:** haploidentical stem cell transplantation (HSCT), peripheral blood stem cells (PBSCs), anti-thymocyte globulin (ATG), post-transplantation cyclophosphamide (PTCy), acute leukemia (AL), Graft versus host disease (GvHD)

## Abstract

In haploidentical peripheral blood stem cell transplantation (haplo-PBSCT), the combination of anti-thymocyte globulin and post-transplant cyclophosphamide (ATG/PTCy) has a synergistic impact in preventing graft-versus-host disease (GvHD). However, little is known about the long-term consequences of the new combination approach. Our goal is to evaluate the efficacy of ATG/PTCy versus a standard ATG regimen by focusing at long-term outcomes in a more homogeneous group of patients. We retrospectively included 118 adult patients up to 60 years with acute leukemia who underwent haplo-PBSCT at our single institution, following the same myeloablative conditioning regimen. From 2010 to 2020, 78 patients received a modified combination of ATG (2.5 mg/kg/day, on days −3, −2, and −1) and PTCy (40 mg/kg/day on days +3 and +4) compared to 40 patients who had a standard ATG-based regimen (2.5 mg/kg/day from days −4 to −1) from 2008 to 2015. The median follow-up time for all patients was 5.36 years, respectively. The cumulative incidence (CI) of neutrophil and platelet engraftment, as well as CMV reactivation, did not differ statistically between the two groups. The CI of the acute GvHD of grades II–IV and III–IV and extensive chronic GvHD were considerably lower in the ATG/PTCy (34.6%, 8.97%, and 13.63%) than in the ATG cohort (57.5%, 30%, and 38.23%) as validated by multivariable modeling. Additionally, compared to the ATG arm, the ATG/PTCy was a hazard factor associated with a higher risk of relapse (HR = 2.23, p = 0.039). The probability of 5-year overall survival, disease-free survival, and GvHD-free relapse-free survival in the ATG/PTCy group (53.34%, 49.77%, and 36.04%) was comparable with the ATG group (47.5%, 42.5%, and 22.5%), respectively. Our finding suggested that a modified ATG/PTCy combination resulted in a lower risk of acute and chronic GvHD and a higher risk of relapse than the standard ATG-based protocol but had no effect on long-term outcomes. However, certain adjustments in the immunosuppression protocol are warranted to improve the outcome.

## Introduction

Despite significant advancements in hematopoietic stem cell transplantation (HSCT) methods and supporting measures in recent years, one of the fundamental challenges facing HSCT institutions is locating a suitable alternative donor at the optimal time when an HLA-matched sibling donor is unavailable (1, 2). Significant improvements in haploidentical (haplo)-HSCT results comparable to matched donor transplants, the ability to rapidly identify an available and affordable donor, and the possibility of repeat stem cell harvesting have contributed to a significant increase in the utilization of haplo-HSCT (3–5). In recent years, the use of two distinct graft-versus-host disease (GvHD) prophylactic methods, including *in vivo* T-cell reduction with anti-thymocyte globulin (ATG) and immunological tolerance promotion with high-dose post-transplant cyclophosphamide (PTCy), seems to have a great potential to enhance haplo-HSCT results (6, 7).

The G-CSF/ATG regimen (Beijing Protocol) with a total ATG dose of 10 mg/kg was initially employed for haplo-HSCT in the context of MAC and a mix of bone marrow stem cells (BMSCs) and peripheral blood stem cells (PBSCs) as a graft source (6). ATG can enhance engraftment and diminish rejection by depletion recipient T-cells (8), so it is now widely used in various transplant types, including unrelated donor HSCT (9, 10). However, it increases the chances of infectious disorders, such as CMV reactivation, due to a delay in immune reconstitution (11).

To overcome HLA barriers in mismatched HSCT, the Baltimore group established a PTCy-based regimen of 50 mg/kg on days +3 and +4 following a non-MAC regimen and BMSC infusion (7). PTCy administration early after graft infusion reduces GvHD by attenuating quickly growing alloreactive T-cells following antigen exposure. Interestingly, regulatory T-cells, which play an important role in preventing GvHD, are resistant to PTCy due to increased aldehyde dehydrogenase production (12, 13).

The significant reduction in alloreactivity caused by a PTCy-based protocol raises concerns regarding recurrence and graft failure (12, 14). Therefore, some institutes have applied the PTCy with a MAC regimen and PBSC source to minimize the chance of relapse (15, 16). However, it was suspected that a higher frequency of acute and chronic GVHD would be associated with the source of PBSC than BMSC in haplo-HSCT with PTCy (17, 18).

A new method combining ATG and PTCy with adjusted dosages was developed to reduce GvHD in haplo-HSCT with PBSC (haplo-PBSCT). In comparison to previous protocols, ATG’s removal of early active T-cells combined with PTCy’s suppression of quickly expanding T-cells resulted in a synergistic immunomodulatory strategy that lowered the risk of GvHD in haplo-PBSCT without influencing other outcomes (19–25). Given this, nothing is known about the long-term consequences of the combined approach, and the members of an international expert panel have yet to endorse the use of ATG in PTCy-based haplo-HSCT (26).

The purpose of this study is to compare the long-term outcome of a modified ATG/PTCy combination to those of an ATG-based protocol in a homogenous cohort of adult patients with acute leukemia who underwent haplo-PBSCT, following the same MAC regimen. Our findings might contribute to the current debate over the utility of combined ATG/PTCy for haplo-PBSCT.

## Material and methods

### Data collection and ethical considerations

The ethical committee of the Hematology, Oncology and Stem Cell Transplantation Research Center (HORCSCT), affiliated with Tehran University of Medical Sciences (TUMS), approved the study (IR.TUMS.HORCSCT.REC.1399.011). The work was carried out by relevant guidelines and regulations. All of the patients gave informed and written consent to the transplantation procedure and use of their data.

Patients’ and donors’ demographic, clinical, and laboratory data were collected from their medical profiles using a checklist. We updated the data and followed the patients until late April 2021.

### Study design

Haplo transplantation was accomplished for the first time at our center in 2008, employing an ATG-based protocol in the setting of a PBSC source and busulfan (Bu)-based MAC. After approximately 2 years, PTCy was added to the ATG-based protocol to reduce GvHD even more, and a new regimen combining ATG, PTCy, and cyclosporine A (CyA) was developed as part of a prospective clinical trial (IRCT ID: IRCT201208041030N11) for a group of haplo-HSCT recipients, while the ATG-based protocol (including ATG, CyA, and methotrexate) remained the standard for haplo patients. Due to the advantages of the ATG/PTCy method, it was adopted as the standard regimen for haplo-PBSCT at our facility in 2015.

In the current study, we retrospectively included all consecutive adult patients (n=118) up to 60 years with AML or ALL who underwent haplo-PBSCT following an identical Bu-based MAC regimen at a single high-volume tertiary referral center (HORCSCT). Subjects who got an ATG/PTCy combination (n=78) from 2010 to 2020 were compared with those who received a standard ATG-based regimen (n=40) from 2008 to 2015. To ensure a homogeneous cohort with few confounding variables, patients who received the graft from bone marrow or cord blood were excluded, as were those who underwent a less intense conditioning regimen.

### Donor selection and source of graft

Donors were chosen based on their HLA-A, -B, -C, -DRB1, and -DQB1 allelic levels using high-resolution HLA typing. Haploidentical donors had two or more HLA mismatches and would be considered without an HLA-matched donor or if HSCT was desired urgently. All haplo-HSCT recipients were tested for donor-specific anti-HLA antibodies (DSAs).

In our institution, PBSC is the preferred graft source for adult patients receiving allo-HSCT for acute leukemia; all patients in the current study received unmanipulated granulocyte-colony stimulating factor (G-CSF)-mobilized PBSC. The median number of CD34+ cells requested was 4 × 10^6^ CD34+/kg of recipient body weight.

### Transplant procedure

All recipients of haplo-HSCT received the same MAC consisting of Bu at 4 mg/kg/day oral or Bu (Busilvex) at 3.2 mg/kg/day i.v. on days −6 through −3, and cyclophosphamide 60 mg/kg/day on days –3 and –2. The GvHD prophylaxis consisted of CyA started at a dose of 1.5 mg/kg/day i.v. on day −2, followed by 3 mg/kg/day from +7 until oral tolerance, rabbit ATG Thymoglobulin; Genzyme, Lyon, France) 2.5 mg/kg i.v. on days –3, –2, and –1, and PTCy 40 mg/kg/day i.v. on days +3 and +4, in the combined ATG/PTCy protocol (27), whereas in the standard ATG-based regimen, patients received ATG at a total dose of 10 mg/kg (2.5 mg/kg/day from − 4 to – 1), CSA, and methotrexate 10 mg/m^2^ on day +1, followed by 6 mg/m^2^ on days +3, +6, and +11.

All patients were given the same prophylactic regimens to prevent herpes simplex virus, candida, and Pneumocystis jirovecii, which contained acyclovir, fluconazole, and trimethoprim/sulfamethoxazole. Screening for cytomegalovirus (CMV) reactivation using DNA polymerase chain reaction was done twice weekly, and in the cases of CMV reactivation, preemptive therapy with ganciclovir was administered.

In our center, there is no routine preventive strategy for avoiding post-transplantation recurrence. However, preemptive intervention with hypomethylating medications (HMAs), FLT3 inhibitors, and repeated escalation doses of donor leukocyte infusion (DLI) were implemented for AML patients who exhibited impaired donor chimerism or positive MRD during post-transplant follow-up. 

### Outcomes and definitions

All outcomes were assessed from the time of transplantation, including OS, DFS, GRFS, non-relapse mortality (NRM), relapse, the acute GvHD (aGvHD) of grade II–IV and III–IV, extensive chronic GvHD (cGvHD), CMV reactivation, platelet (PLT), and absolute neutrophil count (ANC) engraftment. OS was referred to be a time to die. DFS was the length of time after transplantation during which no disease was found. GRFS was defined as survival without evidence of grade III–IV aGVHD, extensive cGVHD requiring systemic immunosuppressive treatment, or recurrence (28). NRM was defined as death without relapse. ANC engraftment was defined as having an ANC of ≥500 cells/μl for 3 days in a row. PLT recovery was characterized by a constant PLT count of more than 20 × 109/L without the need for transfusion in the previous 7 days. The cumulative incidence (CI) of aGvHD (grade II–IV, grade III–IV) and extensive cGvHD were measured 100 days and 1 year following HSCT, respectively. To diagnose and grade aGvHD and cGvHD, Glucksberg’s and the National Institutes of Health consensus criteria were employed (29, 30).

We used the disease risk index (DRI) to risk-stratify patients based on the remission status and cytogenetics and confined it to two risk categories: low/intermediate and high/very high (31). The remission status at HSCT was used to categorize patients as having either the first remission (CR1), relapsed refractory (R/R) disease (defined as the second or more remission and the first remission of primary refractory disease), and active disease was defined by the failure to achieve remission (bone marrow blasts <5%), following induction chemotherapy.

### Statistical analysis

All demographic, clinical, and laboratory characteristics were compared between two groups (ATG and ATG/PTCy) using the Mann–Whitney test for continuous variables and the chi-square test for categorical variables. Median follow-up time was calculated by the reverse Kaplan–Meier method. OS, DFS, and GRFS were estimated by the Kaplan–Meier method and compared among different categories of each covariate using the log-rank χ² test. The CIs of PLT recovery, ANC recovery, CMV reactivation, aGvHD II–IV, aGvHD III–IV, extensive cGvHD, relapse, and NRM were calculated and compared by the Fine and Gray tests.

The Cox proportional hazard regression model was used for the univariable and multivariable analyses of OS, DFS, and GRFS. The proportionality assumption of hazards was tested for each covariate, using Schoenfeld’s residuals and plotting criteria. The univariable and multivariable Fine and Gray proportional subdistribution hazard regression model was applied to test covariates’ associations with relapse, NRM, and GvHD incidences.

The age of the recipient and donor, DRI, Karnofsky Performance Score (KPS), sex matching, ABO matching, underline disease, pretransplant remission status, and GvHD prophylaxis protocol (ATG/PTCy vs. ATG) were used as covariates. All variables with a p-value <0.2 in the univariable analyses were incorporated in the multivariable analysis. A significance level of 0.05 was used for all analyses. Analyses were conducted using Stata (version 11.2, Stata Corp LP, College Station, TX, USA) and packages “survival”, “cmprsk”, and “coxphf” in R software version 3.3.1.

## Results

### Patient characteristics

The research population included 118 consecutive adult patients who underwent haplo-PBSCT at our single center. Seventy-eight patients got a combined ATG/PTCy regimen, while 40 patients received an ATG-based protocol. **Table 1** summarizes the baseline characteristics of both research groups. Apart from the median donor age, recipient CMV status, and donor–recipient sex matching, no other statistically significant difference existed between the two groups.

**Table 1:** Baseline demographic, clinical, and laboratory characteristics of patients and their comparative evaluation according to treatment groups.

		ATG + PTCy	ATG	P-value
**Total, N (%)**		78 (66.1)	40 (33.9)	—
**Recipient age (y) median (IQR)**		27.5 (19–34)	23 (19–34)	0.42
**Donor age (y) median (IQR)**		33 (24–41)	39.5 (30–49)	**0.014**
**Days from diagnosis to HSCT** **median (IQR)**		358 (239–,629)	424 (204–676)	0.87
**Disease Status at HSCT** **N (%)**	**First remission**	25 (32)	9 (22.5)	0.13
	**Relapsed/refractory**	48 (61.5)	24 (60)	
	**No remission**	5 (6.4)	7 (17.5)	
**Primary Disease** **N (%)**	**ALL**	24 (30.77)	16 (40)	0.41
	**AML**	54 (69.23)	24 (60)	
**Sex Matching** **N (%)**	**F to F**	10 (12.8)	9 (22.5)	**0.044**
	**F to M**	26 (33.3)	19 (47.5)	
	**M to F**	11 (14.1)	6 (15)	
	**M to M**	31 (39.7)	6 (15)	
**ABO Matching** **N (%)**	**Matched**	48 (61.54)	21 (52.5)	0.32
	**Minor**	15 (19.23)	7 (17.5)	
	**Major**	13 (16.67)	8 (20)	
	**Bidirectional**	2 (2.56)	4 (10)	
**Recipient CMV Serostatus**	**Positive**	64 (83)	33 (97)	**0.041**
**KPS** **N (%)**	**≥80**	63 (82.9)	31 (79.5)	0.65
	**60–80**	13 (17.1)	8 (20.5)	
**DRI** **N (%)**	**Low/intermediate**	39 (52.7)	16 (43.2)	0.34
	**High/very high**	35 (47.3)	21 (56.7)	
**Graft Cell Dose Median (IQR)**	**CD3^+^ (×10^6^/kg)**	317.5 (249–387)	334 (285–379.5)	0.27
	**CD34^+^ (×10^6^/kg)**	6.1 (4–8.4)	5.43 (3.93–7.26)	0.24

ATG, anti-thymocyte globulin; PTCy, post-transplant cyclophosphamide; ALL, acute lymphoblastic leukemia; AML, acute myeloid leukemia; F, female; M, male; KPS, Karnofsky Performance Score; DRI, disease risk index. P-values significant at the 0.05 level are bold.

All of the patients were followed for a median of 5.36 years. The median follow-up time for ATG/PTCy and ATG was 4.5 (95% CI: 3.5–5.1) and 8.2 (95% CI: 5.9–9.5) years. During follow-up, 52.5% of patients (n=62) died with 48.7% in the ATG/PTCy group and 60.0% in the ATG group, respectively, and 27 patients (22.9%) relapsed, with 19 (24.35%) in ATG/PTCy and the rest (n=8, 20.0%) in the ATG arm. In the ATG/PTCy and ATG arms, the median time to relapse after transplantation was 9.26 and 12.68 months, whereas the median time to NRM was 3.19 and 5.08 months.

### Transplant characteristics

The cumulative incidences of ANC engraftment on day 30 (88.4% vs. 95.0%, P=0.74) and PLT engraftment on day 90 (89.74% vs. 95.0%, P=0.65) did not differ between the ATG/PTCy and ATG groups. The median time to ANC recovery was equal in ATG/PTCy (17 days) and ATG (16 days) groups (P=0.58), although there was a trend for a longer median time to PLT recovery in the ATG/PTCy arm (23 days) versus the ATG arm (18 days) (P = 0.064).

At day 30 post-HSCT, 114 patients with successful myeloid engraftment exhibited complete donor chimerism. The details of donor chimerism after 3, 6, and 12 months following transplantation are provided in **Supplementary Table 1**.

The cumulative incidence of CMV reactivation at 100 days was comparable between ATG/PTCy and ATG (70% vs. 69.2%; P=0.96). The causes of post-transplant mortality are summarized in **Table 2** of the supplement.

**Table 2:** Univariable and multivariable modeling for grade III–IV acute graft-versus-host disease (GvHD) and extensive chronic GvHD.

		Acute GvHD III–IV				Chronic GvHD extensive			
		Univariable		Multivariable		Univariable		Multivariable	
		HR (CI %)	P-value	HR (CI %)	P-value	HR (CI %)	P-value	HR (CI %)	P-value
**Patient Age**		0.99 (0.96–1.02)	0.856	—		0.97 (0.94–1.00)	0.081	0.98 (0.95–1.01)	0.259
**Donor Age**		1.06 (1.03–1.09)	<0.0001	1.07 (1.03–1.12)	**<0.0001**	1.04 (1.01–1.06)	0.001	1.03 (1.00–1.06)	**0.025**
**Arm**	**ATG**	Ref.	—	Ref.	—	Ref.	—	Ref.	—
	**ATG/PTCy**	0.25 (0.13–0.49)	<0.0001	0.25 (0.13–0.49)	<0.0001	0.30 (0.16–0.54)	<0.0001	0.34 (0.17–0.66)	0.002
**Primary Disease**	ALL	Ref.	—	Ref.	—	Ref.	—	Ref.	—
	AML	0.68 (0.36–1.30)	0.250	—		0.76 (0.42–1.39)	0.390	—	
**Remission Status**	**First Remission**	Ref.		Ref.		Ref.	—	Ref.	—
	**R/R**	1.68 (0.76–3.71)	0.192	1.16 (0.47–2.84)	0.738	1.96 (0.97–3.97)	0.059	2.28 (0.93–5.59)	0.071
	**No remission**	0.68 (0.14–3.18)	0.627	0.164 (0.03–0.79)	**0.025**	0.71 (0.15–3.19)	0.658	0.59 (0.09–3.75)	0.584
**Sex Matching**	**F to F**	Ref.	—	Ref.	—	Ref.	—	Ref.	—
	**F to M**	0.69 (0.31–1.51)	0.361	0.41 (0.14–1.17)	0.097	9.07 (2.12–38.74)	0.003	9.02 (1.69–47.98)	**0.01**
	**M to F**	0.62 (0.22–1.72)	0.361	0.66 (0.20–2.14)	0.492	1.24 (0.16–9.08)	0.832	1.60 (0.20–12.81)	0.65
	**M to M**	0.17 (0.05–0.54)	0.003	0.24 (0.07–0.75)	**0.015**	0.99 (0.17–5.52)	0.991	1.36 (0.22–8.54)	0.73
**ABO Matching**	**Matched**	Ref.	—	Ref.	—	Ref.	—	Ref.	—
	**Minor**	2.57 (1.31–5.04)	0.006	2.77 (1.38–5.56)	**0.004**	1.25 (0.59–2.62)	0.554	1.76 (0.84–3.69)	0.132
	**Major**	0.87 (0.33–2.23)	0.775	1.02 (0.32–3.28)	0.963	0.73 (0.34–1.58)	0.435	0.52 (0.20–1.29)	0.160
**KPS**	**≥80**	Ref.	—	Ref.	—	Ref.	—	Ref.	—
	**60–80**	1.18 (0.55–2.52)	0.665	—		0.75 (0.26–2.17)	0.602	—	
**DRI**	**Low/Intermediate**	Ref.	—	Ref.	—	Ref.	—	Ref.	—
	**High/very High**	0.94 (0.49–1.80)	0.868	—		0.61 (0.33–1.13)	0.121	0.38 (0.18–0.82)	**0.014**

ATG, anti-thymocyte globulin; PTCy, post-transplant cyclophosphamide; ALL, acute lymphoblastic leukemia; AML, acute myeloid leukemia; R/R, relapsed/refractory; F, female; M, male; KPS, Karnofsky Performance Score; DRI, disease risk index; GvHD graft-versus host disease; HR, hazard ratio; CI, confidence interval. P-values significant at the 0.05 level are bold.

More than 90% of recipients and donors tested positive for Epstein–Barr virus (EBV) at the time of transplantation. EBV reactivation was seen in 12 (15.38%) and 5 (12.5%) participants in the ATG/PTCy and ATG groups, respectively. Only one patient in the ATG/PTCy arm was found to have post-transplant lymphoproliferative disease (PTLD) as evidenced by biopsy. At our facility, preemptive rituximab therapy for PTLD was not commonly administered. Hemorrhagic cystitis of grades 2–4 related with the BK virus was detected in 26 (33.3%) and 11 (27.5%) patients in the ATG/PTCy and ATG groups, respectively.

Fifteen (12.7%) patients exhibited a non-infectious fever on the first post-transplantation day and reacted swiftly to supportive interventions, implying the probability of low-grade cytokine release syndrome (CRS). ATG-mediated *in vivo* T-cell depletion might cause a lower prevalence and severity of CRS in our haplo-PBSC patients. Moderate-to-severe VOD was recorded in 14 (17.94%) and 6 (15%) ATG/PTCy and ATG patients, respectively.

### Acute and chronic graft-versus-host disease

**Figure 1:**
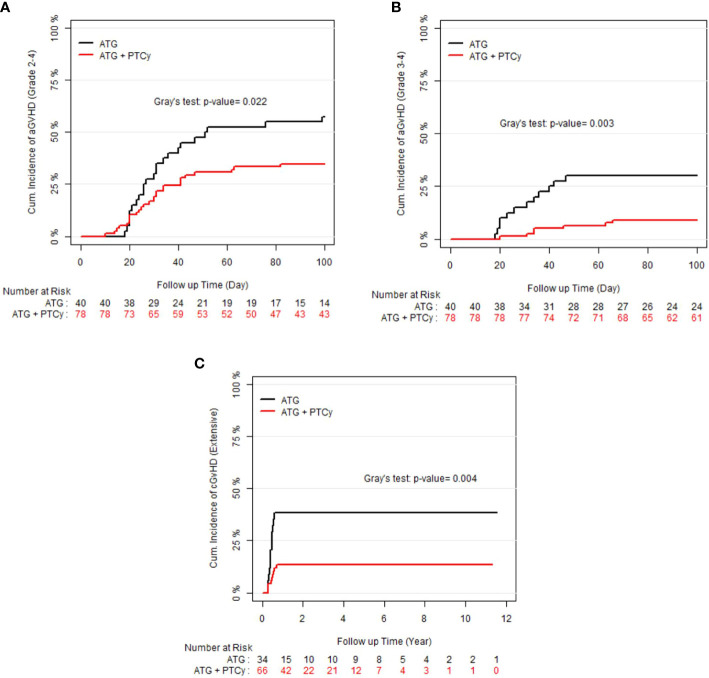
Cumulative incidence (CI) of graft-versus-host disease (GvHD) according to treatment groups; **(A)** CI of grade II–IV acute GvHD; **(B)** CI of grade III–IV acute GvHD; **(C)** CI of extensive chronic GvHD.

As shown in the **Figures 1A–C**, the 100-day CI of grade II–IV and III–IV aGvHD, as well as 1-year extensive cGvHD, was significantly lower in the ATG/PTCy group [34.6% (24.23–45.2), 8.97% (3.09–21.74), and 13.63% (6.65–23.10)] compared to the standard ATG group [57.5% (40.45–71.28), 30% (16.61–44.58), and 38.23% (21.99–54.31)] and confirmed by multivariable modeling. As presented in **Table 2**, the multivariable modeling of GvHD revealed that the ATG/PTCy combination was an independent protective factor that declined the risk of grade III–IV aGvHD by approximately 75% (HR = 0.25; P<0.0001) and extensive cGvHD by nearly 65% (HR = 0.34; P = 0.002) compared to standard ATG-based regimens.

A minor ABO mismatch compared to ABO matched (HR = 2.77; P = 0.004) and advanced donor age (HR= 1.07, P0.0001) were also associated with a higher risk of grade III–IV aGvHD, whereas active disease at transplant compared to first remission (HR=0.164, P=0.025) and male to male compared with female to female (HR=0.24, P=0.015) were associated with lower aGvHD (grade III–IV). When the donor and recipient were mismatched as female to male, the risk of extensive cGvHD increased nine-fold [HR=9.02, P=0.01] compared with female to female.

### Overall survival, disease-free survival, and graft-versus-host disease-free relapse-free survival

**Figure 2:**
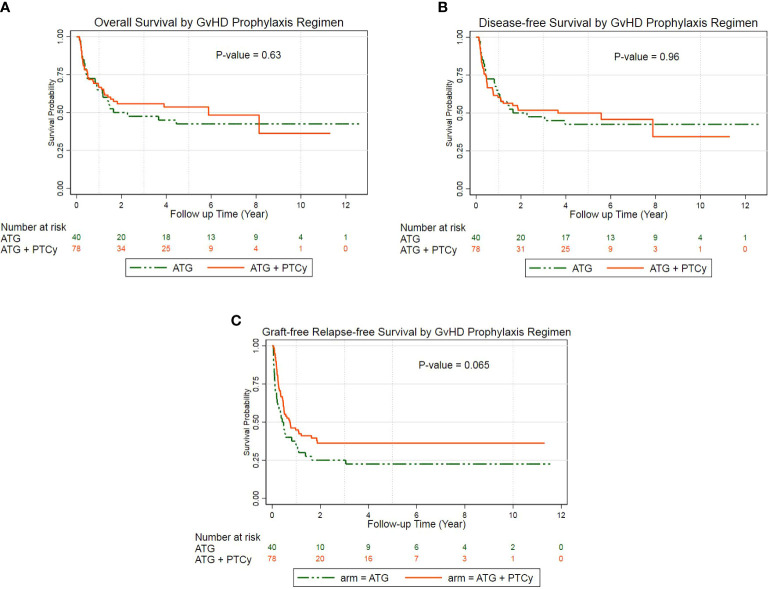
Probability of survival according to treatment groups: **(A)** Kaplan–Meier estimates of overall survival; **(B)** Kaplan–Meier estimates of disease-free survival; **(C)** Kaplan–Meier estimates of GvHD-free, relapse-free survival.

For the entire population, the median OS, DFS, and GRFS times were 4.44, 3.05, and 0.54 years, respectively. The probability of 5-year OS, DFS, and GRFS in the ATG/PTCy group [53.34% (41.25–60.02), 49.77% (37.89–60.55), and 36.04% (25.36–46.82)], respectively, were comparable to the ATG group [47.5% (31.56–61.84), 42.5% (27.15–57.04), and 22.5% (11.15–36.26)] (**Figures 2A–C**).

The multivariable Cox regression analysis of the outcomes revealed that the combined ATG/PTCy regimen did not affect OS, DFS, or GRFS compared to the ATG regimen. The KPS of 60–80 rather than ≥80 was the key predicted risk factor for OS (HR = 3.81, P = 0.000), DFS (HR = 3.25, P = 0.000), and GRFS (HR = 2.34, P = 0.003), independent of a GvHD prophylactic regimen. For OS and DFS, however, no further significant risk factors appeared; nevertheless, for GRFS, donor age was an independent hazard factor (HR = 1.03, P = 0.001), and male to male was a protective factor compared with female to female (HR = 0.37, P = 0.008) (**Table 3**). Furthermore, AML was a marginally preventative measure for OS, lowering the risk of death by more than half compared to ALL (HR: 0.48, P = 0.056).

**Table 3:** Univariable and multivariable modeling for relapse incidence and GvHD-free relapse-free survival.

		Relapse				GRFS			
		Univariable		Multivariable		Univariable		Multivariable	
		HR (CI %)	P-value	HR (CI %)	P-value	HR (CI %)	P-value	HR (CI %)	P-value
**Patient Age**		0.98 (0.96–1.01)	0.363	—	—	1.00 (0.98–1.02)	0.95	—	—
**Donor Age**		1.00 (0.98–1.02)	0.617			1.04 (1.02–1.06)	<0.0001	1.03 (1.01–1.05)	**0.001**
**Arm**	**ATG**	Ref.	—	Ref.	—	Ref.	—	Ref.	—
	**ATG/PTCy**	1.65 (0.89–3.07)	0.110	2.23 (1.04–4.77)	**0.039**	0.65 (0.41–1.03)	0.067	0.90 (0.55–1.46)	0.67
**Primary Disease**	**ALL**	Ref.	—	Ref.	—	Ref.	—	Ref.	—
	**AML**	0.36 (0.21–0.63)	<0.0001	0.25 (0.13–0.49)	**<0.0001**	0.64 (0.41–1.01)	0.056	0.75 (0.46–1.22)	0.25
**Remission Status**	**First remission**	Ref.	—	Ref.	—	Ref.	—	Ref.	—
	**R/R**	0.66 (0.36–1.23)	0.196	0.45 (0.21–0.95)	0.138	1.93 (1.12–3.31)	0.016	1.49 (0.84–2.64)	0.196
	**No remission**	2.88 (1.30–6.36)	0.009	2.39 (0.80–7.15)	0.117	2.42 (1.11–5.27)	0.026	1.01 (0.42–2.43)	0.97
**Sex Matching**	**F to F**	Ref.	—	Ref.	—	Ref.	—	Ref.	—
	**F to M**	0.87 (0.40–1.88)	0.740	0.63 (0.24–1.67)	0.358	0.87 (0.47–1.59)	0.65	0.71 (0.38–1.34)	0.30
	**M to F**	0.87 (0.33–2.27)	0.777	0.65 (0.21–2.01)	0.461	0.54 (0.24–1.20)	0.134	0.58 (0.25–1.33)	0.20
	**M to M**	0.43 (0.17–1.07)	0.071	0.39 (0.14–1.07)	0.068	0.40 (0.20–0.79)	0.008	0.37 (0.18–0.77)	**0.008**
**ABO Matching**	**Matched**	Ref.	—	Ref.	—	Ref.	—	Ref.	—
	**Minor**	1.89 (0.99–3.61)	0.051	1.51 (0.64–3.53)	0.338	1.41 (0.80–2.48)	0.23	—	
	**Major**	1.57 (0.815–3.03)	0.177	1.35 (0.58–3.13)	0.477	1.36 (0.80–2.31)	0.243	—	
**KPS**	**80**	Ref.	—	Ref.	—	Ref.	—	Ref.	—
	**60–80**	0.87 (0.40–1.89)	0.742	—		2.71 (1.59–4.61)	<0.0001	2.34 (1.32 – 4.13)	**0.003**
**DRI**	**Low/intermediate**	Ref.	—	Ref.	—	Ref.	—	Ref.	—
	**High/very high**	2.02 (1.11–3.67)	0.021	1.55 (0.70–3.42)	0.270	1.28 (0.81–2.02)	0.287	—	

ATG, anti-thymocyte globulin; PTCy, post-transplant cyclophosphamide; ALL, acute lymphoblastic leukemia; AML, acute myeloid leukemia; R/R, relapsed/refractory; F, female; M, male; KPS, Karnofsky Performance Score; DRI, disease risk index; GRFS, GVHD-free, relapse-free survival; HR, hazard ratio; CI, confidence interval. P-values significant at the 0.05 level are bold.

### Relapse and non-relapse mortality

**Figure 3:**
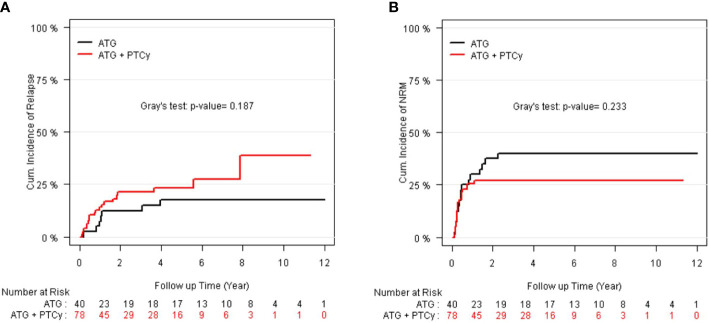
CI of relapse and non-relapse mortality (NRM) according to treatment groups: **(A)** CI of relapse; **(B)** CI of NRM.

As illustrated in **Figures 3A, B**, the 5-year relapse incidence [23.13% (14.9–33.51), 17.5% (7.53–30.88); P = 0.187] and 5-year NRM [26.92% (17.58–37.12), 40.0% (24.72–54.83); P = 0.233] for ATG/PTCy and standard ATG groups were comparable. However, as shown in **Table 3**, the multivariable modeling of relapse revealed that, compared to the ATG arm, the ATG/PTCy was a hazard factor associated with a higher risk of relapse (HR = 2.23, p = 0.039). In contrast, AML, compared to ALL, was associated with a lower risk of relapse (HR=0.25, p <0.0001).

The multivariable analysis of NRM indicated that the GvHD prophylaxis regimen did not influence the risk of NRM (HR = 0.87, p = 0.604). However, increasing donor age (HR = 1.03, p = 0.003) and KPS of 60–80 rather than ≥80 (HR=3.96, p <0.0001) were significantly associated with higher NRM.

### Treatment of post-transplantation relapse

Nine (31%) of the 29 patients with post-transplantation hematologic relapse did not accept any therapeutic intervention for relapse, while five (17.24%) patients with AML gained intensive chemotherapy with FLANG (fludarabine + high-dose cytarabine + mitoxantrone + G-CSF) followed by repeated (two-to-five infusions) escalating-dose DLI in combination with or without an HMA. Seven patients (24.13%) with relapsed ALL underwent rigorous chemotherapy with FLAG preceded by a DLI, whereas the rest eight patients (27.58%) received a single-agent therapy such as HMAs, low-dose cytarabine, vincristine, and FLT3 inhibitors. None of our patients had a second allo-HSCT transplant.

### Cause of death

As presented in **Supplementary Table 2**, of the 62 deaths, relapse was the major cause for the entire population (n = 27, 43.5%), accounting for 50.0% and 33.3% in the ATG/PTCy and ATG groups, followed by infectious diseases (38.7%). GvHD was the cause of death in 4 out of 24 patients (16.7%) in the ATG group, although not in the combined cohort.

## Discussion

As previously indicated, the propensity toward adopting PBSC to avoid recurrence in the haplo-HSCT felt the need for a more potent GvHD prophylaxis regimen, driving some institutions to propose a combined strategy (ATG/PTCy). A few studies have sought to study the effect of concurrent ATG/PTCy in the haplo-PBSCT and have mainly employed adjusted dosages to reduce side effects. This study contributes to the body of knowledge by examining long-term outcomes in a more homogeneous group of patients.

In **Table 4**, we summarized the results of previous publications that employed a combined (ATG and PTCy) regimen for haplo-PBSCT. As shown in **Table 4**, various forms of the combination protocol have been designed, including low-dose ATG (2.5 mg/kg total) plus standard-dose PTCy (21), standard-dose ATG (10 mg/kg total) in conjunction with low-dose PTCy (14.5 mg/kg, days +3 and +4) (25), half doses of both ATG (5 mg/kg, total) and PTCy (50 mg/kg, total) (23), and finally, our work that a modified dose of both ATG (7.5 mg/kg, total) and PTCy (80 mg/kg, total) was combined. It is concluded that despite the different strategies, the ATG/PTCy protocol was generally associated with a considerable decrease in all kinds of GvHD without altering other outcomes compared to an ATG- or PTCy-based regimen.

**Table 4:** Summary of previous publications that employed a combined (anti-thymocyte globulin and post-transplant cyclophosphamide) regimen for haploidentical peripheral blood stem cell transplantation.

Study type	ATG and PTCy compared to ATG									ATG and PTCy			Single arm
										Compared to PTCy			ATG and PTCy
**Reference**				(23)			(25)			(21)			(20)
**Articles**	**Our study**			**Xu X, et al. BMT 2021**			**Yu Wang, et al.** **J Hem and Oncol. 2019**			**Makanga DR, et al.** **Jimmunol 2020**			**Salas MQ, et al. BMT.2020**
	**1-year**			**1-year**			**2-year**			**1-year**			**1-year**
**Stem cell sources**	**PBSC**			**PBSC and CBU**			**PBSC**			**PBSC**			**PBSC**
**Conditioning regimen**	MAC (Bu/Cy)			MAC or RIC (BFA)			MAC			RIC (CloB2A1)	RIC Baltimore	P.v	RIC (TFB)
**Study arm**	ATG and PTCy (N=78)	ATG (N=40)	P.v	ATG and PTCy (N=31)	ATG (N=36)	P.v	ATG and PTCy (N=114)	ATG (N=125)	P.v	ATG and PTCy (N=26)	PTCy (N=32)		ATG and PTCy (N=52)
**Total dose of ATG and PTCy**	7.5 and 80 (mg/kg)	10 mg/kg	5 and 50 (mg/kg)	10 mg/kg	10 and 29 (mg/kg)		10 mg/kg	2.5 and 100 (mg/kg)		100 mg/kg	4.5 and 100 (mg/kg)	
**OS**	66.67%	65.00%	0.63	74.90%	59.40%	0.25	83.00%	77.00%	0.18	61.50%	71.50%	0.75	58.80%
**DFS**	60.26%	62.50%	0.96	64.20%	55%	0.4	81.00%	71.00%	0.06	53.80%	59.30%	0.98	53.30%
**GRFS**	48.72%	37.50%	0.065	—	—	—	63.00%	48.00%	**0.02**	53.80%	43.70%	0.24	37.80%
**Acute GvHD II–IV**	34.60%	57.50%	**0.022**	17.00%	40.20%	**0.042**	26.00%	36.00%	0.14	23.80%	59.30%	**0.004**	21.20%
**Acute GvHD III–IV**	8.97%	30.00%	**0.003**	3.20%	23.10%	**0.025**	5.00%	18.00%	**0.003**	4.70%	18.70%	0.29	4.60%
**Chronic GvHD extensive**	13.63%	38.23%	**0.004**	11.20%	40%	**0.029**	17.00%	16.00%	0.71	11.00%	20.60%	0.65	—
**Relapse incidence**	14.10%	7.50%	0.18	23.00%	8.70%	**0.042**	13.00%	14.00%	0.62	30.70%	37.50%	0.79	31.00%
**NRM**	25.64%	30.00%	0.23	12.90%	36.20%	**0.038**	6.00%	15.00%	**0.045**	19.20%	18.70%	1	31.00%
**Common cause of death**	Relapse	Infection	—	Relapse	GvHD	—	Relapse	Infection	—	Relapse	Relapse	—	Infection
**DRI; high/very high**	44.90%	52.50%	NS	51.60%	61%	NS	12.00%	17.00%	NS	42.30%	25.00%	NS	27.00%
**CMV reactivation**	69.20%	70.00%	0.96	42.00%	63.80%	0.072	74.00%	30.00%	**<0.001**	—	—	—	77.00%

ATG, anti-thymocyte globulin; PTCy, post-transplant cyclophosphamide; DRI, disease risk index; OS, overall survival; DFS, disease-free survival; GRFS, GvHD-free, relapse-free survival; GvHD, graft vs. host disease; NRM, non-relapse mortality; PBSC, peripheral blood stem cell; CB, cord blood; MAC, myeloablative conditioning; RIC, reduced intensity conditioning; P.V, P-value. P-values significant at the 0.05 level are bold.

In terms of long-term OS and DFS, consistent with other studies (23, 25), our findings revealed no significant differences between ATG/PTCy- and ATG-based protocols and were confirmed by multivariable models. Similar to Xu X et al. (23), our results demonstrated the significantly lower aGvHD and extensive cGvHD in the ATG/PTCy compared to the standard ATG group, whereas this is true only for grades of III–IV aGvHD in Yu Wang et al. (25) and grade II–IV aGvHD in Makanga DR et al. (21).

After adjustment by multivariable analysis, the risk of relapse was considerably greater in the ATG/PTCy group than in the standard ATG group (**Table 3**), as confirmed by Xu X et al. (23); however, as shown in **Table 4**, Yu Wang et al. (25) demonstrated a similar relapse incidence between two arms, possibly due to a lower-dose PTCy in the combined protocol. However, the 1-year CI of relapse for the ATG/PTCy group in our research (14.10%) was lower than that reported by Makanga DR et al. (21) and Salas MQ, et al. (20) for the combined regimen in haplo-PBSCT with reduced-intensity conditioning (RIC).

We found no significant differences in NRM and GRFS between the two groups; however, Xu X et al. (23) found a significantly lower NRM for ATG/PTCy, and Yu Wang et al. (25) found a significantly bigger GRFS for the combination of ATG with low-dosage PTCy compared to the standard ATG group. Furthermore, the disease status was shown to be a clinical risk factor that increased the risk of NRM by more than twofold (HR=2.05, P=0.09) for R/R patients compared to the first remission, even though it did not attain statistical validity. The greater 3-year NRM in the R/R group compared to the CR1 group (39.4% vs. 17.6%) was mostly due to the R/R group’s increased infectious comorbidities.

Finally, we observed that, regardless of GvHD prophylactic regimen, a KPS of 60–80, rather than 80, was the main predictive risk factor for OS, DFS, and GRFS, increasing the risk of mortality mostly through worsening NRM. Additionally, as shown in previous studies (32, 33), older donor age was found to be a significant predictor of outcomes that adversely affect GRFS and NRM by raising the risk of both aGVHD (grade III–IV) and extensive cGvHD; hence, it is advised to take this into account when selecting donors.

There are various restrictions and advantages to this research. We conducted to evaluate two GvHD-preventive regimens in a homogenous group of adult patients with acute leukemia who had haplo-PBSCT using the same Bu-based MAC regimen, the same graft source, and the same transplant protocol in a single facility with as few confounding factors as feasible.

The retrospective character of our investigation, as well as its single-center design and limited sample size, all contributed to its limitations. The absence of data on immune reconstitution, as well as incomplete information on cytogenetic or molecular evaluations such as the FLT3 or NPM1 mutational status in certain patients, made it tough to determine the initial risk stratification or do conventional MRD surveillance following transplantation.

## Conclusion

In this retrospective cohort of adult patients with acute leukemia who received haplo-PBSCT after Bu-based MAC, we found that dual *in vivo* TCD with a modified combination of ATG and PTCy resulted in a lower risk of acute and chronic GvHD and a higher risk of relapse compared to the standard ATG-based protocol but had no effect on long-term outcomes in terms of OS, DFS, GRFS, and NRM.

Although, given that infectious diseases were the second leading cause of death and the main cause of NRM in our study population, as well as the high incidence of post-transplant CMV reactivation (roughly 70% for both arms) due to ATG-induced T-cell depletion (34), future immunosuppressive protocol modifications or the use of a less-severe conditioning regimen, particularly for R/R patients, as well as the adoption of a more effective CMV-preventive regimen like letermovir, seem to have the potential to enhance the result (35).

In this regard, we have just started prospective randomized research to see if early ATG administration during conditioning (2.5 mg/kg on days –9, –8, and –7) in conjunction with PTCy reduces the risk of post-transplant infections such as CMV reactivation when compared to our standard combination approach (IRCT20140818018842N20). In addition, the other clinical research is presently ongoing in our facility to assess a new CMV prophylaxis regimen for our haplo recipients, including a combination of valaciclovir and ganciclovir instead of acyclovir that was used in the previous protocol.

## Data availability statement

The raw data supporting the conclusions of this article will be made available by the authors, without undue reservation.

## Ethics statement

The ethical committee of the Hematology, Oncology and Stem Cell Transplantation Research Center (HORCSCT), affiliated with Tehran University of Medical Sciences (TUMS), approved the study (reference IR.TUMS.HORCSCT.REC.1399.011). The patients provided their written informed consent to participate in this study.

## Author contributions

MB and AK conceptualized and organized the study; MB wrote the text; AK analyzed the data and wrote the results. All authors participated to collecting data and approval process of the manuscript. All authors approved the submitted version.

## Funding

The authors would like to thank the Research Institute for Oncology, Hematology and Cell Therapy (RIOHCT), Tehran University of Medical Sciences (TUMS), Tehran, Iran, for their financial support through small grant (TUMS, Grant No. 98-3-107-45357).

## Acknowledgments

We want to thank our colleagues for clinical management and our data registry team for providing data to the study.

## Conflict of interest

The authors declare that the research was conducted in the absence of any commercial or financial relationships that could be construed as a potential conflict of interest.

## Publisher’s note

All claims expressed in this article are solely those of the authors and do not necessarily represent those of their affiliated organizations, or those of the publisher, the editors and the reviewers. Any product that may be evaluated in this article, or claim that may be made by its manufacturer, is not guaranteed or endorsed by the publisher.
