# Supplementary material for: Modified combination of anti-thymocyte globulin (ATG) and post-transplant cyclophosphamide (PTCy) as compared with standard ATG protocol in haploidentical peripheral blood stem cell transplantation for acute leukemia
Source: Front Immunol. 2022 Aug 5;13:921293. doi: 10.3389/fimmu.2022.921293 (PMC9388846; doi:10.3389/fimmu.2022.921293)
Supplement: Supplementary file 1 [file Table_1.docx]

| **Supplementary Table 1** The number (%) of patients with the full or impaired donor chimerism at 3, 6, and 12 months after transplantation based on the study’s groups | | | | | | | | | | | |  |  |  |  |  |
| --- | --- | --- | --- | --- | --- | --- | --- | --- | --- | --- | --- | --- | --- | --- | --- | --- |
|  | **chimerism day +90** | | | **chimerism day +180** | | | | **chimerism day +365** | | | |  |  |  |  |  |
|  | **< 95%** | **≥ 95%** | **Total** | **< 95%** | **≥ 95%** | **not evaluated** | **Total** | **< 95%** | **≥ 95%** | **not evaluated** | **Total** |  |  |  |  |  |
| **ATG/PTC** | 5 (7.46%) | 62 (92.53%) | 67 (100%) | 6 (10.52%) | 47 (82.45%) | 4 (7.01%) | 57 (100%) | 8 (15.68%) | 32 (62.74%) | 11 (21.56%) | 51 (100%) |  |  |  |  |  |
| **ATG** | 1 (2.85%) | 34 (97.14%) | 35 (100%) | 0 (0%) | 26 (89.65%) | 3 (10.34%) | 29 (100%) | 4 (15.38%) | 16 (61.53%) | 6 (23.07%) | 26 (100%) |  |  |  |  |  |
| **Total** | 6 (5.88%) | 96 (94.11%) | 102 (100%) | 6 (6.97%) | 73 (84.88%) | 7 (8.13%) | 86 (100%) | 12 (15.58%) | 48 (62.33%) | 17 (22.07%) | 77 (100%) |  |  |  |  |  |
|  | | | | | | | | | | | |  |  |  |  |  |
|  |  |  |  |  |  |  |  |  |  |  |  |  |  |  |  |  |
|  |  |  |  |  |  |  |  |  |  |  |  |  |  |  |  |  |
|  |  |  |  |  |  |  |  |  |  |  |  |  |  |  |  |  |
|  |  |  |  |  |  |  |  |  |  |  |  |  |  |  |  |  |
|  |  |  |  |  |  |  |  |  |  |  |  |  |  |  |  |  |
|  |  |  |  |  |  |  |  |  |  |  |  |  |  |  |  |  |
