# Supplementary material for: Modified combination of anti-thymocyte globulin (ATG) and post-transplant cyclophosphamide (PTCy) as compared with standard ATG protocol in haploidentical peripheral blood stem cell transplantation for acute leukemia
Source: Front Immunol. 2022 Aug 5;13:921293. doi: 10.3389/fimmu.2022.921293 (PMC9388846; doi:10.3389/fimmu.2022.921293)
Supplement: Supplementary file 2 [file Table_2.docx]

| Supplementary Table 2 The number (%) of post-HSCT events | | | | |
| --- | --- | --- | --- | --- |
| Arm | | **ATG/PTC** | **ATG** | **Total** |
| Acute GvHD 2-4 | | 27 | 22 | 49 |
|  |  | 34.60% | 55.00% | 41.50% |
| Acute GvHD 3-4 | | 7 | 12 | 19 |
|  |  | 9.00% | 30.00% | 16.10% |
| Ext chronic GvHD | | 9 | 12 | 21 |
|  |  | 11.5% | 30.0% | 17.8% |
| CMV reactivation | | 54 | 28 | 82 |
|  |  | 69.2% | 70.0% | 69.5% |
| EBV reactivation | | 12 | 5 | 17 |
|  |  | 15.4% | 12.5% | 14.4% |
| Post HSCT Relapse | | 21 | 8 | 29 |
|  |  | 26.9% | 20.0% | 24.6% |
| Couse of death | **Relapse** | 19 | 8 | 27 |
|  |  | 50.00% | 33.30% | 43.50% |
|  | **GvHD** | 0 | 4 | 4 |
|  |  | 0.00% | 16.70% | 6.50% |
|  | **Infection** | 15 | 9 | 24 |
|  |  | 39.50% | 37.50% | 38.70% |
|  | **Primary Graft Failure** | 3 | 1 | 4 |
|  |  | 7.90% | 4.20% | 6.50% |
|  | **Secondary Graft Failure** | 1 | 2 | 3 |
|  |  | 2.60% | 8.30% | 4.80% |
| Total Death | | 38 | 24 | 62 |
|  |  | 48.70% | 60.00% | 52.50% |
| Total | | 78 | 40 | 118 |
|  |  | 100.00% | 100.00% | 100.00% |
